# Generation of “Virtual” Control Groups for Single Arm Prostate Cancer Adjuvant Trials

**DOI:** 10.1371/journal.pone.0085010

**Published:** 2014-01-21

**Authors:** Zhenyu Jia, Michael B. Lilly, James A. Koziol, Xin Chen, Xiao-Qin Xia, Yipeng Wang, Douglas Skarecky, Manuel Sutton, Anne Sawyers, Herbert Ruckle, Philip M. Carpenter, Jessica Wang-Rodriguez, Jun Jiang, Mingsen Deng, Cong Pan, Jian-guo Zhu, Christine E. McLaren, Michael J. Gurley, Chung Lee, Michael McClelland, Thomas Ahlering, Michael W. Kattan, Dan Mercola

**Affiliations:** 1 Department of Statistics, University of Akron, Akron, Ohio, United States of America; 2 Division of Hematology-Oncology, Medical University of South Carolina, Charleston, South Carolina, United States of America; 3 College of Health, Human Services and Science, Ashford University, California, United States of America; 4 Department of Pathology & Laboratory Medicine, University of California Irvine, Irvine, California, United States of America; 5 Institute of Hydrobiology, Chinese Academy of Sciences, Wuhan, China; 6 AltheaDx Inc., San Diego, California, United States of America; 7 Department of Urology, University of California Irvine, Irvine, California, United States of America; 8 Department of Urology, Loma Linda University, Loma Linda, California, United States of America; 9 Department of Pathology, University of California San Diego, La Jolla, California, United States of America; 10 Guizhou Provincial Key Laboratory of Computational Nano-Material Science, Guizhou Normal College, Guiyang, China; 11 Department of Urology, Guizhou Provincial People's Hospital, Guizhou, China; 12 Department of Epidemiology, University of California Irvine, Irvine, California, United States of America; 13 Department of Urology, Northwestern University Feinberg School of Medicine, Chicago, Illinois, United States of America; 14 Department of Medicine, University of California Irvine, Irvine, California, United States of America; 15 Vaccine Research Institute of San Diego, San Diego, California, United States of America; 16 Department of Quantitative Health Sciences, Cleveland Clinic, Cleveland, Ohio, United States of America; 17 Department of Family and Community Medicine, Northeast Ohio Medical University, Rootstown, Ohio, United States of America; Texas Tech University Health Sciences Center, United States of America

## Abstract

It is difficult to construct a control group for trials of adjuvant therapy (Rx) of prostate cancer after radical prostatectomy (RP) due to ethical issues and patient acceptance. We utilized 8 curve-fitting models to estimate the time to 60%, 65%, … 95% chance of progression free survival (PFS) based on the data derived from Kattan post-RP nomogram. The 8 models were systematically applied to a training set of 153 post-RP cases without adjuvant Rx to develop 8 subsets of cases (reference case sets) whose observed PFS times were most accurately predicted by each model. To prepare a virtual control group for a single-arm adjuvant Rx trial, we first select the optimal model for the trial cases based on the minimum weighted Euclidean distance between the trial case set and the reference case set in terms of clinical features, and then compare the virtual PFS times calculated by the optimum model with the observed PFSs of the trial cases by the logrank test. The method was validated using an independent dataset of 155 post-RP patients without adjuvant Rx. We then applied the method to patients on a Phase II trial of adjuvant chemo-hormonal Rx post RP, which indicated that the adjuvant Rx is highly effective in prolonging PFS after RP in patients at high risk for prostate cancer recurrence. The method can accurately generate control groups for single-arm, post-RP adjuvant Rx trials for prostate cancer, facilitating development of new therapeutic strategies.

## Introduction

Prostatectomy provides excellent disease control for the majority of patients with clinically-localized prostate cancer. However, for patients at a high risk of relapse, additional (adjuvant) therapy may be needed to prevent disease recurrence. Enrolling control groups in early-phase exploratory studies of novel adjuvant regimens is problematic due to ethical issues and patient acceptance. The comparison of new treatments with historical controls can yield biased results, because differences in patient selection can easily confound the findings. The best control would be the patients themselves if they were not treated with adjuvant therapy. Therefore, an alternative to concurrent or historical control groups may be to construct a “virtual” control group for a set of patients by estimating progression-free survival (PFS) based on their post-radical prostatectomy (post-RP) clinical characteristics. The estimated PFS for the virtual control group will be compared to the observed PFS for the treated group using the logrank test [1], [2] to evaluate the efficacy of therapy. Such controls would likely be more closely matched to the study subjects than would a set of historical controls that only approximated the characteristics of the study population. Thus, the key step for generating a predicted control group is to estimate the PFS times based on the patients' post-RP clinical characteristics.

Predictive nomograms in oncology are graphical representations of mathematical formulae or algorithms that incorporate observations for relevant clinical characteristics in order to predict a particular end point. Such nomograms are typically based on traditional statistical methods such as multivariable logistic regression or Cox proportional hazards analysis [3]–[5]. The “Kattan” nomogram originally was presented in 1999 [6], and was updated in 2005 [7] and 2009 [8]. These nomograms utilize patient-specific parameters to calculate a series of probabilities of being progression-free at various times following prostatectomy. All versions are similarly accurate in predicting the chance of post-RP PFS, with concordance-indices between 0.7680 and 0.7859 [9]. Although nomograms have been used to estimate PFS probabilities at arbitrary times [10], the available online version of the Kattan post-RP nomogram [6] only provides PFS *probabilities* for each patient at a number of time points, e.g., at years 2, 5, and 7, after surgery. These discrete Kattan probability values cannot be used for logrank analysis; they need to be converted first to a single time measurement for each patient.

Here we present a novel method involving 8 models for converting the Kattan probability values to estimated time measurements, with each model represented by a reference case set that has a different level of relapse risk. Trial cohorts with higher relapse risk require higher stringency models. The optimal model is selected for the trial cohort by carefully matching the 8 reference case sets to the trial cohort based on the patients' post-RP clinical characteristics.

## Materials and Methods

### Patient datasets

#### Training and validation datasets

Radical prostatectomy cases for training and validation were identified from the authors' practices and research databanks. These subjects had received no form of adjuvant or salvage therapy. All datasets used a PSA threshold of >0.2 ng/mL, or the new appearance of radiographic lesions consistent with metastases, for definition of relapse. Frequency of radiographic and PSA monitoring was at the discretion of the treating physicians. We have obtained approval from UC Irvine IRB. Written consent was given by the patients including their information normally stored in the hospital database to be used for research. High-risk cases exhibited one or more of the following characteristics: 1) pre-operative PSA >15 ng/mL, 2) Gleason score ≥8, 3) extraprostatic extension, 4) invasion of seminal vesicles, 5) lymph node metastases, 6) positive surgical margins, 7) persistently detectable PSA ≥0.2 ng/mL more than 45 days after surgery. Most cases had two or more of these features. For each source of training and validation datasets, all cases that met the necessary definitions, and that had all relevant data, were utilized. Required data included type of operation, date of operation, pre-operative PSA level, age at surgery, prostatectomy Gleason score, seminal vesicle status, lymph node status, margin status, extraprostatic extension status, one or more PSA values ≥45 days after surgical date, relapse status, date of relapse status assessment, and at least 1 year of followup time.

We created a training set of 153 prostate cancer cases consisting of patients with a wide range of relapse risks following radical prostatectomy. The majority consisted of 112 RP cases at the Long Beach VA Hospital (Long Beach, CA) from December 1990 to June 1998. To increase the proportion of medium- and high-risk cases we added 41 cases from the UCI SPECS registry of 1,220 cases. The SPECS (Strategic Partners for the Evaluation of Cancer Signatures) consortium project was an NIH/NCI-funded study that sought to identify predictive biomarkers for early relapse after prostatectomy [11]–[15].

A validation dataset of 155 cases was constructed with 62 cases from the University of California, Irvine (UCI), 32 cases from Loma Linda University (LLU), and 62 additional cases from the UCI SPECS registry used exclusively for validation (SPECS(2)). None of these later SPECS cases (SPECS(2)) had been used in the training dataset. Because we anticipated use of our method with single-arm adjuvant therapy studies, we used only medium- and high-risk cases in the validation set to mimic the likely population that would be involved in such studies. Characteristics of the training and validation sets can be found in **Table 1**.

**Table 1:** Characteristics of Patient Cases.

Characteristic	Training set	Test set	Matched set	Adjuvant set
Number	153	155	20	20
Age at prostatectomy	65.7	64.2	66.5	63
Median Preoperative PSA (ng/ml)	10.25	9.5	15.4	8.4
Surgery				
Open RP	133 (86.9%)	43 (27.7%)	13 (65%)	19 (95%)
Robot-assisted laparoscopic RP	19 (12.4%)	112 (72.3%)	7 (35%)	0
Open radical cystoprostatectomy	0	0	0	1 (5%)
Unknown	1 (0.7%)	0	0	0
Years of surgery	1990–2009	2000–2011	1992–2009	2000–2006
Lymph Node Status				
N0	137 (89.5%)	136 (87.7%)	6 (30%)	4 (20%)
N1	16 (10.5%)	19 (12.3%)	14 (70%)	15 (75%)
Insufficient data	0	0	0	1 (5%)
Extraprostatic Extension				
No	66 (43.1%)	27 (17.4%)	8 (40%)	6 (30%)
Yes	87 (56.9%)	128 (82.6%)	12 (60%)	13 (65%)
Insufficient data	0	0	0	1 (5%)
Surgical margins Positive				
No	72 (47.1%)	87 (56.1%)	9 (45%)	9 (45%)
Yes	81 (52.9%)	68 (43.9%)	11 (55%)	11 (55%)
Seminal Vesicles Invasion				
No	131 (85.6%)	93 (60%)	13 (65%)	12 (60%)
Yes	22 (14.4%)	62 (40%)	7 (35%)	7 (35%)
Insufficient data	0	0	0	1 (5%)
Gleason Score				
2–6	36 (23.5%)	13 (8.4%)	1 (5%)	2 (10%)
7 (3+4)	61 (39.9%)	54 (34.8%)	5 (25%)	2 (10%)
7 (4+3)	25 (16.3%)	35 (22.6%)	5 (25%)	4 (20%)
8–10	31 (20.3%)	53 (34.2%)	9 (45%)	12 (60%)

#### Adjuvant therapy dataset

Between 2001 and 2006, 20 subjects with high-risk prostate cancer were treated with open RP followed by adjuvant multimodality therapy (HR, MBL) [16]. All subjects were at high risk of recurrence of prostate cancer, based on one or more of the following clinical features: pT3 or pT4 disease (80%), Gleason score 8–10 (60%), extraprostatic extension (65%), positive surgical margins (55%), tumor in seminal vesicles (35%) or lymph nodes (75%), or high preoperative PSA level (>15 ng/mL; 40%). Patients received docetaxel and estramustine therapy according to the regimen of Petrylak, et al [17], for a median of six cycles, beginning shortly (median 2 months) after surgery. They also received concurrent androgen deprivation therapy (ADT) for a median of 4.3 years. Subjects were monitored for disease recurrence by serial measurement of PSA levels, as well as by standard clinical parameters. Time to relapse was defined as the time from surgery to the first PSA level of 0.2 ng/mL or greater after the chemotherapy treatment component. These patients have been followed for a median of 7.5 years, with a maximum of 11.0 years.

### Nomogram

A web-application (http://www.mskcc.org/cancer-care/adult/prostate/prediction-tools) based on the 1999 Kattan nomogram [6] was used to calculate the PFS probabilities at years 2, 5 and 7 after prostatectomy based on clinical variables including age, margin status, tumor stage, Gleason primary score, Gleason secondary score, pre-op PSA level, seminal vesicle status, lymph node status, and year of prostatectomy.

### Statistical methods

For each patient, we fitted the discrete Kattan PFS probability values at years 0 (assumed to be 100%), 2, 5, and 7 after RP with a Loess curve [18], [19] or Spline [20] (see details in **Supplement** and **Supplementary Figure S1 in File S1**). The fitted curve was used to estimate the time to variable endpoints, *i.e.*, time to 10%, 15%, …, or 95% chance of survival, here termed *model*s (model.10, model.15, …, model.95). In this study, we only used 8 models, all above median risk, *i.e.* model.60, model.65, model.70, model.75, model.80, model.85, model.90, and model.95 because we are interested primarily in studies involving patients at high risk for recurrence.

The key step for our method is the selection of the appropriate model for a particular set of trial cases (treated patients). We initially expected that model.50 (time to 50% chance of survival) would be optimum. However, this model was inadequate to predict PFS, especially for higher-risk case cohorts. We therefore explored additional models with higher stringency. We identified subsets of the training cases (termed reference case sets) for each of the 8 models, where the observed PFS times are most closely predicted by each specific model. The trial cases were then compared to the 8 reference sets based on the similarity of clinical characteristics to determine the best model for generating virtual controls for the trial cases.

#### Construction of reference sets

**Figure 1:**
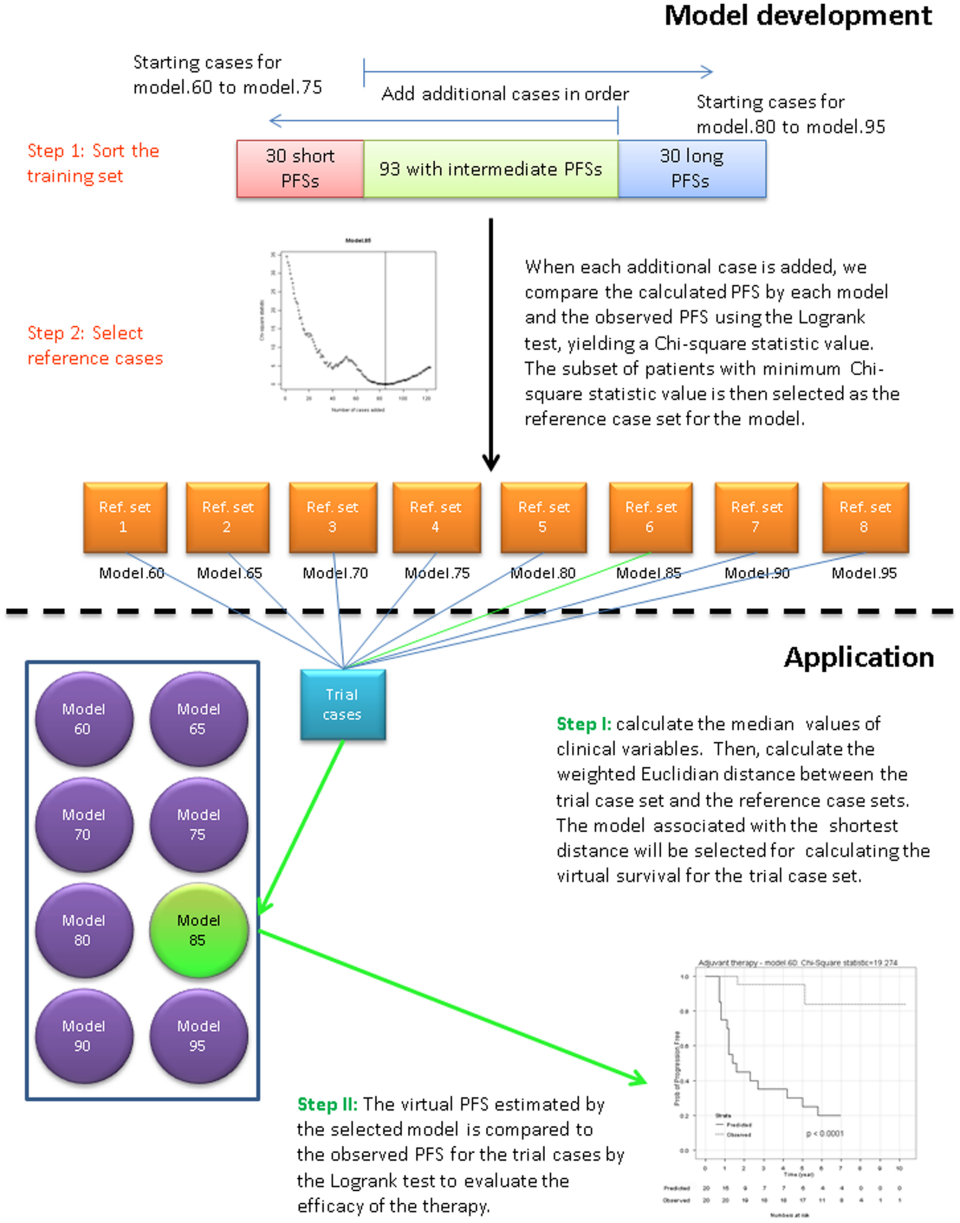
Overall scheme for the development of the method and its application.

**Figure 2:**
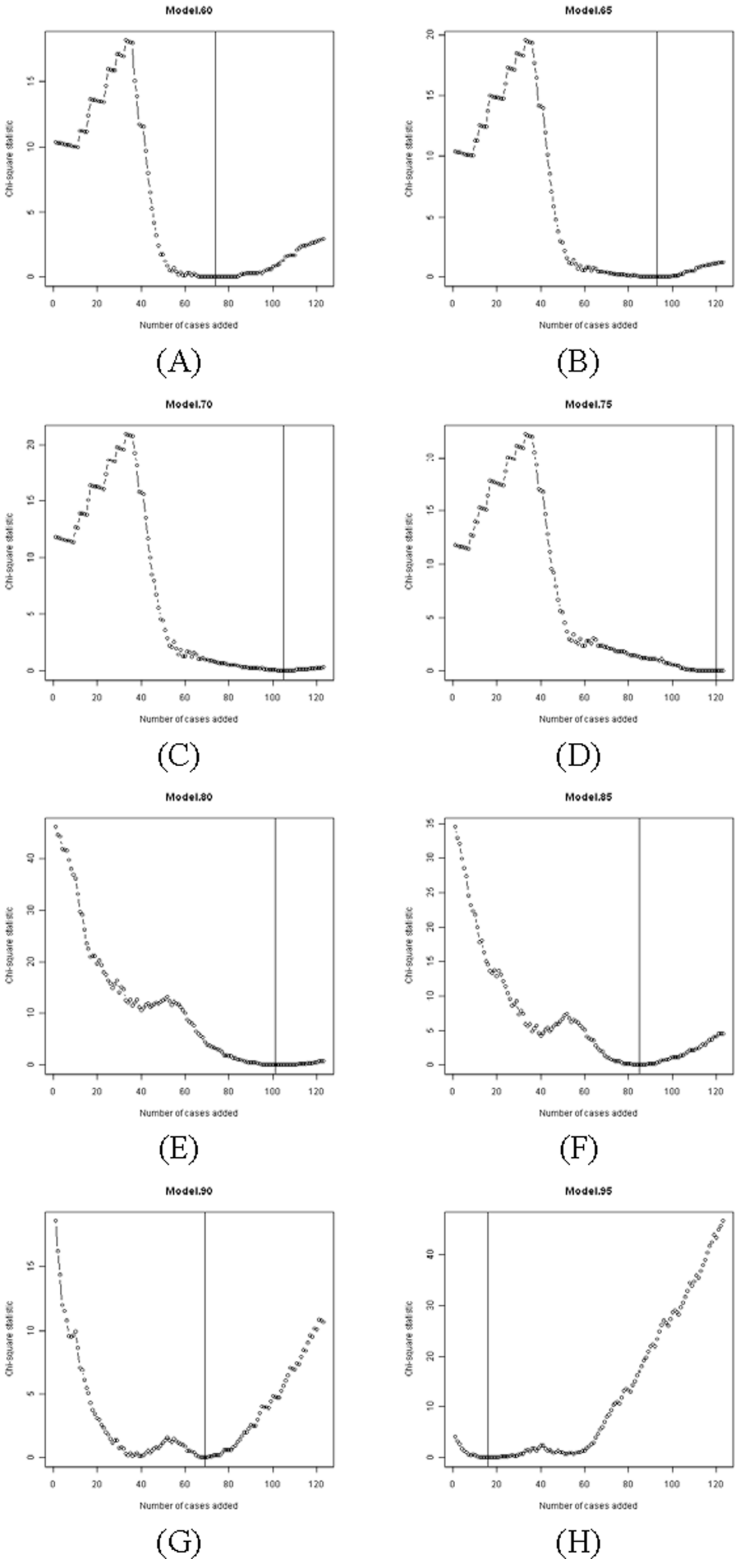
Systematic selection of patient subsets from training set to form 8 reference case sets for the 8 models.

The process of constructing 8 reference sets for the 8 models is depicted in **Figure 1** (upper part). The 153 training cases (adjuvant therapy-free cases) were sorted from short PFS time to long PFS time based on the observed PFS outcomes for these patients. For each model (model.60, model.65, … model.95), we began with a starting subset of 30 cases and then added additional cases in order from the ranked pool of training cases, until all 153 cases had been utilized. As each additional case was added, we repeatedly calculated a PFS comparator group using each of the 8 models, and compared these calculated PFS times to the actual PFS times. The agreement between the observed and calculated PFS times for a subset of the training cases was quantitatively evaluated by the Chi-square statistic of the logrank test. If the calculated PFS times generated by the model agreed with the actual PFS times, the two Kaplan-Meier curves should superimpose. The Chi-square statistic from the logrank test would then be smaller than 3.84 which translates to a p value≥0.05 in Chi-square distribution with degree of freedom 1. However, ifthe two Kaplan-Meier curves would separate, the Chi-square statistics would be greater than 3.84. For any model, a subset of cases that produced the minimum Chi-square statistic would have the optimum clinical characteristics for the use with that model. Therefore, for each model, the Chi-square statistics from the logrank analysis were plotted against the number of added cases (**Figure 2**). The set of cases that produced the minimum Chi-square statistic, indicating maximum agreement of calculated PFS times and observed PFS times, was chosen as “optimum” for that particular model.

#### Selection of the best model

To determine the best model for the treatment trial cases, a series of clinical variables were matched using the weighted Euclidean distance of the clinical parameters, between the trial cases and each set of reference cases (Eqn. 1). Clinical variables considered in distance calculation included age, margin status, pathologic tumor stage, Gleason primary score, Gleason secondary score, pre-op PSA level, seminal vesicle status, lymph node status. We placed more weight on continuous variables than on binary variables in distance calculation, *i.e.*, 17%, 5%, 17%, 17%, 17%, 17%, 5%, 5%, respectively, for these 8 clinical variables. The weighted Euclidean distance based on the 8 clinical variables is defined as:
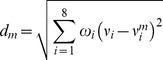
(1)where 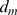 is the weighted Euclidean distance for model *m*, 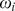 is weight for *i*th clinical variable, 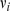 is the median value of the *i*th clinical variable for treatment trial cases, and 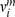 is the median value of the *i*th clinical variables of the *m*th reference case set (*m* = 1, …, 8). The model whose reference cases had the minimum weighted Euclidean distance to the trial cases was then selected for the generation of the control group by estimating the time to relapse (or “virtual” PFS time) for each of the trial cases. The observed PFS times for the trial cases were then compared to the estimated PFS times for those same patients (virtual controls) using the logrank test, to reach a clinical conclusion. The process is depicted in **Figure 1** (lower part).

All the analyses were implemented in the statistical program R (http://www.R-project.org/) and written in R language. A web application for implementing the proposed method is publically available at http://mercola.hs.uci.edu/singlearm/. The overall observed and predicted PFSs for the treated patients were summarized by the Kaplan-Meier method [21]. The logrank test [1], [2] was used to compare Kaplan-Meier curves.

## Results

### Validation using independent test cases

**Figure 3:**
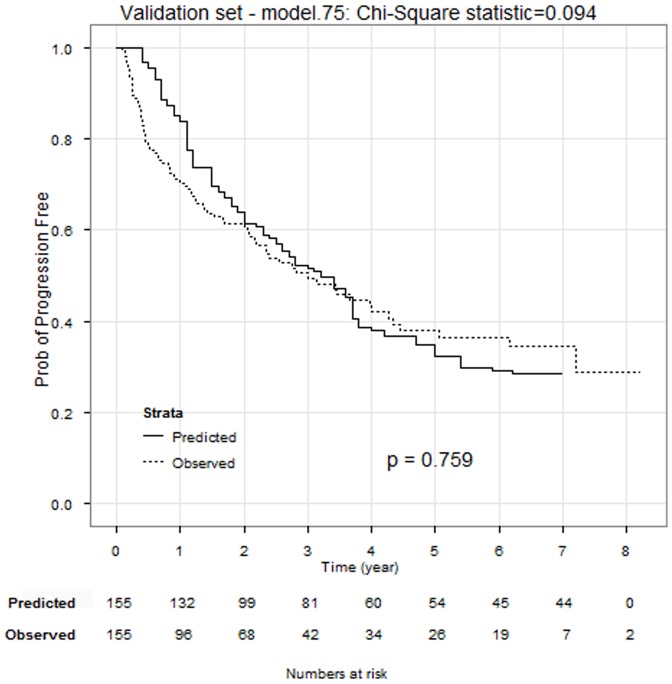
Comparison of predicted PFS with observed PFS in validation set.

To demonstrate the performance of the method, we used a completely independent validation set of 155 cases (**Materials and Methods**). The optimal model (model.75) was identified for the test set. The comparison *via* the logrank test indicated that the predicted PFS times agreed with the observed PFS times very well (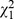 = 0.094 and *p* value>0.05; **Figure 3**).

**Figure 4:**
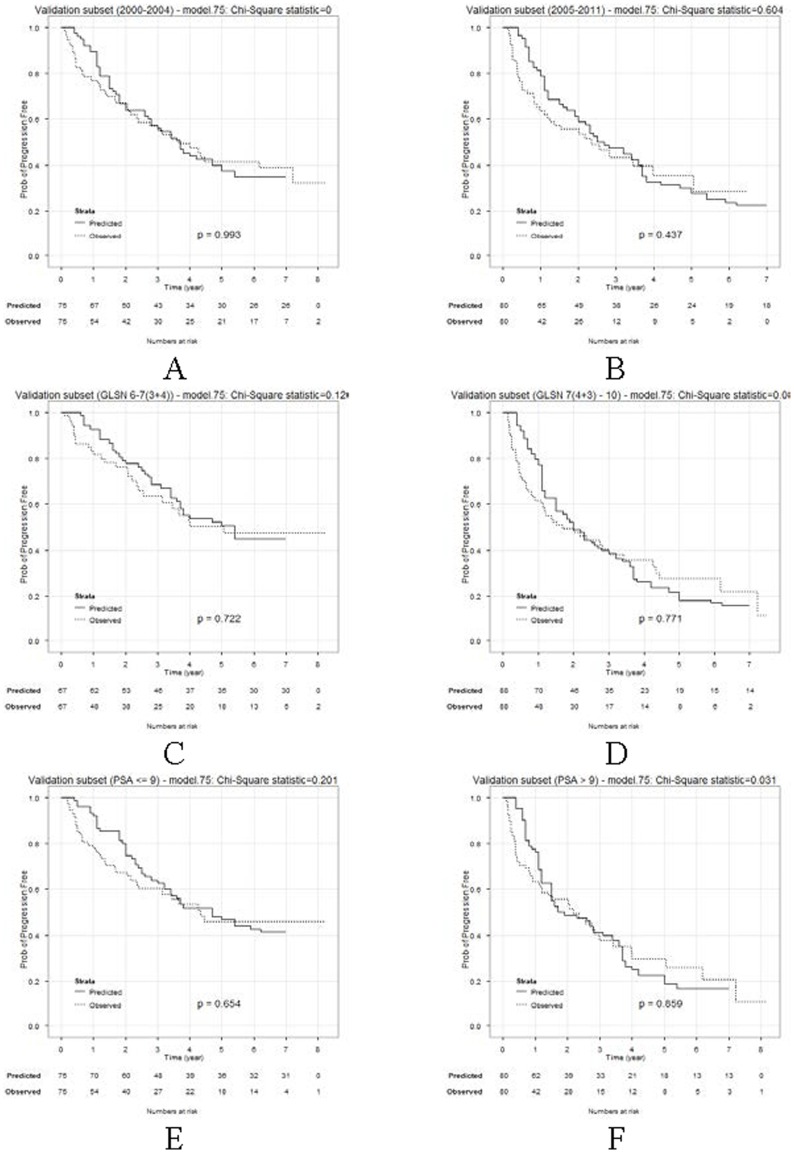
Comparison of predicted PFS with observed PFS in subsets of the validation cohort. Panel A: surgery 2000–2004; Panel B: surgery 2005–2011; Panel C: Gleason score 6–7(3+4); Panel D: Gleason score 7(4+3)–10; Panel E: preoperative PSA ≤9; Panel F: preoperative PSA >9.

To further explore the performance of the method we created 6 smaller validation sets from the 155 validation patients. The first and second subgroups consist of patients who had surgery in years 2000–2004 and years 2005–2011, respectively. The third and fourth subgroups are made up of patients with Gleason score 6–7 (3+4) and 7 (4+3)–10, respectively. The fifth and sixth subgroups represent the patients with initial PSA ≤9 and patients with initial PSA >9, respectively. The comparisons between the observed PFS times and the calculated PFS values *via* the logrank test for these validation sets are summarized in **Figure 4A–4F**. The calculated PFS times agreed with the observed PFS times very well (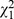
's<3.84 and p values>0.05), demonstrating that the predictive method was robust across a spectrum of clinical characteristics, types of operations, and operation dates.

### Application to adjuvant phase II studies

**Figure 5:**
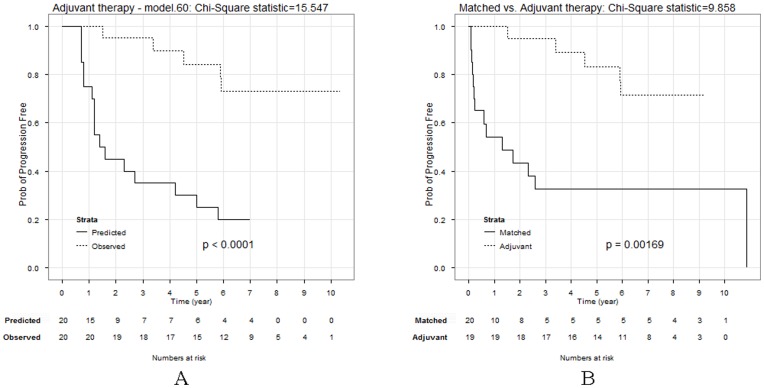
Use of predicted comparator groups in a single-arm adjuvant therapy trial. Panel A: Comparison of predicted and observed PFS for an adjuvant therapy series (n = 20) receiving postoperative chemo-hormonal therapy. Panel B: Kaplan-Meier analysis of PFS for adjuvant therapy patients (n = 20) and matched historical controls (n = 20) from the training set.

We have conducted a phase II study of adjuvant chemotherapy and ADT for subjects at high risk for relapse after radical prostatectomy [16]. To determine if the regimen is active at prolonging PFS, we have used as a comparison group the expected PFS times derived from the aggregate patient Kattan data by the above methods. The matching of eight clinical parameters of our patients with the 8 reference case sets showed that model.60 would be the best model to calculate “virtual” PFS values. By the selected model, we converted the nomogram-predicted probabilities to the estimated PFS time for each of 20 patients (PFS as if they did not received adjuvant therapy) and compared the observed PFSs with the predicted PFSs by the Kaplan-Meier method. The observed PFS significantly differed from the estimated PFS with 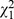 = 19.3 and p value<0.0001 by the logrank test (**Figure 5A**). This comparison had power of 97% to detect a difference in survival given the 10-year survival rates in two groups are 80% and 20% (approximated from Kaplan-Meier curves in **Figure 5**), respectively. The power calculation in various scenarios based on the simplified Rubenstein's formula [22], [23] is given in Supplemental **Table S1 in File S1**, indicating that our analysis was adequately powered to detect discordance between the calculated PFSs and the observed PFSs.

As an additional confirmation that this adjuvant therapy regimen is active, we compared the observed PFS with historical controls — a set of 20 clinically-matched cases that were manually selected from the 153 training cases (see **Table 1**). None of these comparators received adjuvant therapy. However, PFS for our treated control group was significantly better than that seen for the historical, clinically-matched subjects from the matched group (**Figure 5B**). In aggregate these data show that the virtual control group method identifies adjuvant therapy regimens that are capable of improving a significant endpoint, PFS.

## Discussion

It is crucial to construct a control group for evaluating the efficacy of an adjuvant post-prostatectomy therapy when enrolling control groups becomes impractical. Comparisons with *historical* controls can yield anomalous results due to sampling bias. Therefore, the best control would be the patients themselves if they were not treated with adjuvant therapy. Nomograms have been used to construct a control arm based on patients' historical data to deal with single-arm (treatment arm only) trials. For example, Gulley et al. used the Halabi nomogram [24] to estimate the median survival for each patient, and then compared the estimated survival to the observed survival (post-treatment survival) using the logrank test [1], [2]. The Halabi nomogram was derived from patients with metastatic castration-resistant prostate cancer, and therefore is inappropriate for post-RP adjuvant therapy studies. Post-prostatectomy nomograms have also been used to generate comparison groups for adjuvant therapy trials. Kibel et al. performed a phase II study of adjuvant docetaxel in high risk patients [25]. In order to compare to the observed PFS, they used a modified version of the Kattan nomogram [6] to predict progression in each patient, and then averaged the probabilities at each progression time across patients [25]. Similar strategy was used in evaluating efficacy and safety of Pertuzumab in a phase II prostate cancer trial [10]. This method applies when nomogram estimation of PFS is available at arbitrary times. Nevertheless, the online version of the Kattan post-RP nomogram [6] only provides PFS *probabilities* for each patient at 3 time points, i.e., years 2, 5 and 7. Thus, new approaches are needed to extend the application of online version of Kattan post-RP nomogram to single-arm trial data. Model-based methods have been proposed for single-arm phase II trial data [26]; however, this approach has been applied only to the situation where single time point is considered, for example, prediction of 2-year survival probability.

Our initial expectation was that model.50 (time to 50% chance of recurrence) would be the optimum model for most trial cases. Unexpectedly, model.50 performance was suboptimal, *i.e.* the calculated PFS times were significantly longer than the observed PFS times, indicating that model.50 (or median PFS) may overestimate the PFS for high risk patients. We therefore studied the impact of clinical features on the performance of 8 additional models (model.60, model.65, model.70, model.75, model.80, model.85, model.90, model.95). In this study, we developed a novel method based on Kattan's nomogram [6] and which allowed precise calculation of the predicted PFS times for trials with distinct patient compositions.

When we constructed reference sets for the 8 models, we had noted that the optimum model for constructing a control group varied based on the clinical characteristics of the cases used. Model.60, model.65, model.70 and model.75 formed one class of models (class 1) which fitted moderate-risk patients. In contrast model.80, model.85, model.90 and model.95 formed another class of models (class 2) that worked better for high-risk patients. This phenomenon likely results from the weighting of variables used in the nomogram calculation algorithm. For the development of reference cases for the models of these two classes, we utilized different starting subsets. For the models in class 1, we started with the first 30 (long-PFS) cases in the training set, and then added cases sequentially in a long-to-short PFS progression until all 153 cases had been utilized. For models in class 2, we started with the last 30 (short-PFS) cases in the training set, and then added cases sequentially in a short-to-long PFS risk progression until all 153 cases had been utilized. The schemes for selection of the starting subset are due to the limited size of the training set. If we selected 30 long-PFS cases as the starting subset for the models in class 2, the curve of Chi-square statistics would increase without reaching a nadir (minimum Chi-square statistics). Similarly, if we selected the last 30 cases (short-PFS cases) as starting subset for the models in class 1, there would not be a nadir for the curve of Chi-square statistics. **Figure 2** presents the population of Chi-square statistics compared with the number of cases used, for each of the 8 models. Note that as we sequentially examined model.60, model.65, model.70, and model.75 (**Figure 2A–2D**) we had to add in more and more short-PFS cases to the initial set of 30 long-PFS cases. The progression continued for the next four models, though we here started with 30 short-PFS cases (**Figure 2E–2H**). For model.80 we had to add a large number of long-PFS cases to minimize the Chi-square statistics. However, for model.95 we added very few cases, with those primarily being short-PFS patients. Note that there may be several pools of cases with characteristics that can be analyzed well by a particular model. These might be represented graphically by broad down-pointing peaks (rather than spikes) of Chi-square statistics, or by multiple discrete nadirs. In all of our examples however there was a discrete “best” patient population (reference set) for a particular model.

In the course of developing reference sets of cases for the 8 models, we utilized the classical logrank test [1], [2] to compare the observed PFS and the “virtual” PFS estimated by different models. The logrank test is widely used in clinical trials to establish the efficacy of a new treatment compared to a control treatment when the measurement is the time to event, such as time to biological recurrence in prostate cancer patients. If censored observations are not present in the data then the Wilcoxon rank sum test [27] should be used instead. The Chi-square statistic with degree of freedom 1 and its associated *p* value can be easily calculated for the Logrank test. Chi-square statistics greater than 3.84 (*p* value<0.05) indicate that there is significant discrepancy between the observed PFS and the calculated PFS; on the contrary, Chi-square statistics less than 3.84 (P value>0.05) is in favor of the null hypothesis which suggests agreement between the observed PFS and the calculated PFS. The Logrank test may not be simply replaced by concordance index [28] or receiver operating characteristic (ROC) curve-based methods [29] because these methods are not appropriate to comparing two groups of time-measurement survival data involving censoring. These alternative statistics are more suitable to situations where a risk-predictive model is established and prediction accuracy needs to be assessed.

The comparisons between predicted and observed PFSs in the training and validation sets used patient databases derived from multiple surgeons using both open and laparoscopic operations, over a 21-year period of time, at multiple institutions, with variable follow-up patterns. In spite of these variables, our method has functioned well to accurately calculate PFS in a large validation case series as well as the subsets of cases chosen based on year of surgery, Gleason scores and initial PSA. However biases could be problematic with smaller series, which are likely to be the norm for pilot adjuvant therapy trials. Intrinsic differences in the type of operation or the skill or the surgeon could lead to skewed results. The historical version of the Kattan nomogram [6] utilized data primarily derived from open prostatectomy cases, whereas laparoscopic cases are more common now. Moreover, it is known that the Kattan nomogram may underestimate the relapse risk in some populations [9], potentially challenging the model assignment in the study. In addition the common use of a PSA threshold ≥0.2 ng/mL for definition of post-prostatectomy relapse may appear to give a poorer PFS than may be predicted by an algorithm based on the Kattan nomogram, which used a PSA threshold of 0.4 ng/mL or more to define relapse. These theoretical concerns may be overcome by using reference sets that are developed from training set of significantly larger size and complexity than used in this report. We are presently engaged in these studies.

Different data sets have varying time of surgery. For example, Ahlering robot cases (UCI) were 2002–2009, Long Beach VA cases were 1990–1998, Loma Linda University (LLU) adjuvant chemo/hormones cases were 2001–2006, LLU robot cases (Ruckle) were 2007–2010, SPECS cases were 2000–2010. In fact, year of prostectomy is an important variable as it account for changes in diagnostic and therapeutic techniques over time. Given enough samples, one can subgroup samples based on year of surgery (categorical variable), and train reference sets within each subgroup. In this way, the effect of time of prostectomy will be well addressed. However, due to the limited size of training samples in the current study, we do not have enough power to identify the effect of time of surgery. Nevertheless, we did test the performance of the current model on patient samples that had surgery during different time frames, i.e., a 2000–2004 and 2005–2011. The model worked very well on both test sets (Figure 4). Advanced model will be developed based on increased sample base.

Because Kattan numbers can be calculated for every patient, there is no difficulty in obtaining a matched comparator group specific for the study population. The application of the new method to our adjuvant phase II study demonstrated that the adjuvant therapy intervention significantly improved PFS in these patients, compared to the PFS expected with no therapy. Such a result would not be entirely surprising because 75% of our subjects had pN1 disease. Adjuvant ADT alone has been shown to significantly improve progression-free, disease-specific, and overall survival in post-prostatectomy subjects with positive lymph nodes [30]. In addition it appears that adjuvant ADT alone is associated with an excellent overall PFS in high-risk post-prostatectomy subjects [31]. Our patients all received adjuvant chemotherapy in addition to ADT, which may have provided a benefit in our pN0 patients and contributed to the overall, highly-significant difference in observed PFS and predicted PFS for our patients.

In differing subject groups, both of adjuvant post-prostatectomy radiation therapy or androgen deprivation can be effective at significantly improving progression-free, disease-specific, or overall survival. However, neither treatment is optimal. Radiation increases side effects such as strictures and incontinence, as well as rectal injuries. Androgen deprivation may be permanent, and leads to a variety of undesirable side effects such as the metabolic syndrome, impotence and erectile dysfunction, and accelerated loss of bone mass. To identify alternative interventions that might be more acceptable as adjuvant therapies, we need methods to rapidly identify a “signal” for a significant end point (such as progression-free survival). The proposed algorithm can be used as a surrogate endpoint for relatively short term single arm trials, to identify interventions worth further investigation in expensive long-term studies. This algorithm can therefore speed the development of adjuvant therapies with novel agents or combinations that may avoid the toxicities of radiation or androgen deprivation.

## Conclusions

In summary, a new method that rigorously defines appropriate virtual control cases for single arm prostate cancer treatment trials has been developed. A web-based application for this method is available at http://mercola.hs.uci.edu/singlearm.

## Supporting Information

File S1 **Supplementary material including Nonlinear curve fitting and estimation of time to relapse, Figure S1 and Table S1.**
(DOC)
