# Supplementary material for: Generation of “Virtual” Control Groups for Single Arm Prostate Cancer Adjuvant Trials
Source: PLoS One. 2014 Jan 21;9(1):e85010. doi: 10.1371/journal.pone.0085010 (PMC3897405; doi:10.1371/journal.pone.0085010)
Supplement: File S1 — Supplementary material including Nonlinear curve fitting and estimation of time to relapse, Figure S1 and Table S1. (DOC) [file pone.0085010.s001.doc]

**Nonlinear curve fitting and estimation of time to relapse**

For each patient, we used the Loess and Spline functions to fit the nomogram-estimated PFS probabilities at years 2, 5 and 7 with non-linear regression curves. We implicitly assumed a PFS of 100% at year 0 for each patient. The PFS probability for any patient decreased monotonically with time. For each patient, we fitted the 4 discrete PFS probability values with a Loess curve. For model.50, the time to 50% chance of PFS was extrapolated from the fitted Loess model, provided that the nomogram-estimated PFS probability was < 50% at year 7 for the patient. In an example, the nomogram-predicted PFS probabilities at years 0, 2, 5 and 7 were 100%, 64%, 36%, and 29%, respectively (**Supplementary Figure S1**). The Loess-estimated time to 50% chance of PFS for this patient was 3.3 years (red dot). The Loess regression may be replaced by Spline which generated a similar value (3.1 years, the blue dot in **Figure S1**). For the patients whose PFS probability > 50% at year 7, the time to 50% chance of PFS could not be estimated because that point was out of the data range. Therefore, the patients whose PFS probability > 50% at year 7 were simply treated as censored data. By these methods we converted nomogram-predicted PFS probabilities to median survival times, which could then be used for standard Kaplan-Meier analyses. The Loess-fitted curve can be used to estimate the time to variable endpoints, *i.e.*, time to 10%, 15%, …, or 95% chance of relapse, termed different models (model.10, model.15, …, model.95.

**Supplement Figure S1:** Model fitting and estimation for a single patient, using non-linear regression methods. The red/blue line is for Loess/Spline method. The red/blue dot represents the estimated time to 50% of chance of PFS for the patient.

**
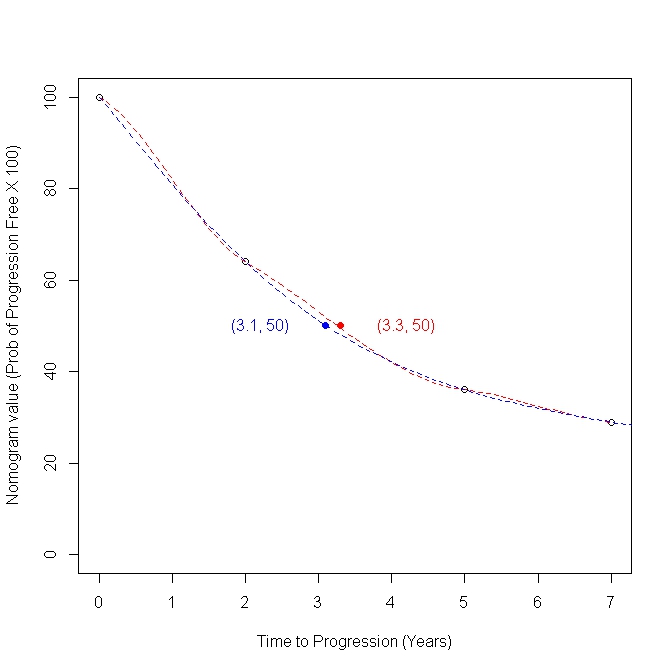
**

**Supplement Table S1:** Power analysis for logrank test. P1 is the survival rate in one group and P2 is the survival rate in the other group. N is the number of cases in each group.

| P1 | P2 | N | Power |  | P1 | P2 | N | Power |
| --- | --- | --- | --- | --- | --- | --- | --- | --- |
| 0.8 | 0.2 | 20 | 0.971 |  | 0.7 | 0.2 | 20 | 0.934 |
| 0.8 | 0.2 | 30 | 0.996 |  | 0.7 | 0.2 | 30 | 0.986 |
| 0.8 | 0.2 | 40 | 1 |  | 0.7 | 0.2 | 40 | 0.997 |
| 0.8 | 0.2 | 50 | 1 |  | 0.7 | 0.2 | 50 | 1 |
| 0.8 | 0.3 | 20 | 0.908 |  | 0.7 | 0.3 | 20 | 0.802 |
| 0.8 | 0.3 | 30 | 0.977 |  | 0.7 | 0.3 | 30 | 0.921 |
| 0.8 | 0.3 | 40 | 0.995 |  | 0.7 | 0.3 | 40 | 0.970 |
| 0.8 | 0.3 | 50 | 0.999 |  | 0.7 | 0.3 | 50 | 0.989 |
| 0.8 | 0.4 | 20 | 0.789 |  | 0.7 | 0.4 | 20 | 0.596 |
| 0.8 | 0.4 | 30 | 0.912 |  | 0.7 | 0.4 | 30 | 0.747 |
| 0.8 | 0.4 | 40 | 0.965 |  | 0.7 | 0.4 | 40 | 0.847 |
| 0.8 | 0.4 | 50 | 0.987 |  | 0.7 | 0.4 | 50 | 0.910 |
